# Agricultural Injuries with Dementia: Double Whammy?

**DOI:** 10.1093/geroni/igaf122.512

**Published:** 2025-12-31

**Authors:** Kanika Arora, Jonathan Davis, Lila Basnet, Julie Bobitt

**Affiliations:** University of Iowa, Iowa City, Iowa, United States; University of Iowa, Iowa City, Iowa, United States; University of Iowa, Iowa City, Iowa, United States; University of Illinois at Chicago, Chicago, Illinois, United States

## Abstract

Older adults make up about one-third of U.S. producers and account for nearly 80% of agricultural fatalities. Moreover, agricultural workers have higher odds of developing dementia, a neurodegenerative disorder with significant safety implications due to impaired judgement, memory loss, and problems with balance, vision, and communication. Farm environments with heavy equipment and livestock further amplify these risks. Despite the fact that older farmers continue to work well into advanced ages, no prior study has examined how dementia impacts agricultural injuries. We examine mechanisms and severity of agricultural injuries with dementia among those 60 years and older using national trauma data from the Trauma Quality Programs Participant Use File (TQP-PUF) over 2017-2021. Using multivariable logistic and ordered logistic regressions, we compare agricultural injuries in individuals with dementia (group 1) to those with agricultural injuries but without dementia (group 2) and those with non-agricultural injuries but with dementia (group 3). Our findings show group 1 has a unique injury profile, combining elements of both agricultural and dementia-related trauma. Compared to group 3, group 1 was less likely to experience falls but more likely to be injured by transport and machinery-related incidents. Alternatively, compared to group 2, group 1 had higher odds of experiencing falls. Group 1 was also more likely to experience “severe” injuries (Injury Severity Score > =16) relative to group 3. Additionally, group 1 members were also more likely to require ICU/surgery at ED Discharge. These results highlight the need for targeted injury prevention programs for older adults with dementia in agriculture.

